# Non-Coding RNAs in Hodgkin Lymphoma

**DOI:** 10.3390/ijms18061154

**Published:** 2017-05-29

**Authors:** Anna Cordeiro, Mariano Monzó, Alfons Navarro

**Affiliations:** Molecular Oncology and Embryology Laboratory, Human Anatomy and Embryology Unit, School of Medicine, University of Barcelona, C/Casanova 143, 08032 Barcelona, Spain; anna.cordeirosa@gmail.com (A.C.); mmonzo@ub.edu (M.M.)

**Keywords:** Hodgkin lymphoma, non-coding RNAs, miRNAs, lncRNAs, piRNAs

## Abstract

MicroRNAs (miRNAs), small non-coding RNAs that regulate gene expression by binding to the 3’-UTR of their target genes, can act as oncogenes or tumor suppressors. Recently, other types of non-coding RNAs—piwiRNAs and long non-coding RNAs—have also been identified. Hodgkin lymphoma (HL) is a B cell origin disease characterized by the presence of only 1% of tumor cells, known as Hodgkin and Reed-Stenberg (HRS) cells, which interact with the microenvironment to evade apoptosis. Several studies have reported specific miRNA signatures that can differentiate HL lymph nodes from reactive lymph nodes, identify histologic groups within classical HL, and distinguish HRS cells from germinal center B cells. Moreover, some signatures are associated with survival or response to chemotherapy. Most of the miRNAs in the signatures regulate genes related to apoptosis, cell cycle arrest, or signaling pathways. Here we review findings on miRNAs in HL, as well as on other non-coding RNAs.

## 1. Introduction

Hodgkin lymphoma (HL), first described in 1832 by the English physician Thomas Hodgkin [1], is one of the most frequent lymphomas in the Western world. It is estimated that 8500 new cases of HL will be diagnosed in 2016 in the USA [2]. The most salient characteristic of HL is that tumor cells constitute less than 1% of the tumor bulk and are surrounded by a protective microenvironment that includes mostly eosinophils, neutrophils, plasma cells, histiocytes, fibroblasts, and stromal cells [3]. The World Health Organization classifies HL in classical HL (cHL), which represents 95% of cases, and nodular lymphocyte predominant HL. cHL in turn has four histological subtypes: nodular sclerosis (NS), mixed-cellularity (MC), and lymphocyte-rich and lymphocyte-depleted [4]. In cHL, tumor cells, known as Hodgkin and Reed-Sternberg (HRS) cells, are mostly derived from germinal center (GC) B cells, while T cell origin has been described in 5–15% of cases [5]. After contacting an antigen, naïve B cells enter the GC, where they participate in the rearrangement of the immunoglobulins VH chain and become either memory B cells or plasma cells. In some cases, however, they can acquire crippling mutations that prevent antigenic selection and apoptosis [6] and become HRS cells [7], which are characterized by the loss of most B cell markers and the acquisition of CD15 and CD30 cell surface markers [8]. Several mechanisms are involved in this evasion of apoptosis, including Epstein-Barr virus (EBV), which is present in 40–60% of HRS cells of cHL in the Western world [9].

Non-coding RNAs (ncRNAs) have emerged as key regulators in normal and pathological cell processes [10]. ncRNAs are RNA molecules transcribed from DNA that are not translated to protein and are classified in two main groups: small ncRNAs, which are less than 200 bp, and long ncRNAs (lncRNAs) [11]. Small ncRNAs include the most widely investigated group, microRNAs (miRNAs), while lncRNAs are still in the early stages of investigation despite the fact that they are more numerous than miRNAs.

miRNAs are endogenous, small, single-stranded molecules from 18 to 25 base pairs. They were discovered in 1993 in the nematode *Caenorhabditis elegans* [12,13], and since then have been identified in several species, including plants [14], viruses [15], and humans [16,17,18]. miRNA biogenesis involves a complex maturation process that begins in the nucleus, where they are transcribed by RNA-polymerase 2 as pri-miRNAs, long molecules that can be over 1 kb and contain several miRNAs. Still in the nucleus, they are processed by the Drosha-DGCR8 complex (Drosha has RNase activity and DGCR8 recognizes the RNA substrate). After processing a ~70 bp autocomplementary molecule, known as pre-miRNA, is produced. They are exported into the cytoplasm by exportin-5 (XPO5) and the Ran-GTP complex, where they are cleaved by a Dicer into a ~22 bp duplex. This duplex binds to the protein complex RISC, which selects one of the strands-the mature miRNA-to guide RISC to the target mRNA, resulting in the inhibition of the target mRNA [17]. Other less frequent functions— dependent and independent of RISC—have also been described for miRNAs [19].

The first evidence that miRNAs were expressed in HL was demonstrated by Kluiver et al. in 2005 [20]. The authors had previously reported a high expression of the BIC gene in HRS cells [21] and found that miR-155 expression correlated with BIC expression in HL cell lines [20]. BIC was identified as a pri-miRNA that can be processed to miR-155 [22]. Since then, several studies have evaluated the role of miRNAs in the pathology and prognosis of HL—both as miRNA signatures and individually.

## 2. miRNA Signatures in HL Tissue and Cell Lines

To date, only six groups have studied miRNA signatures in HL (Table 1), and the evolution of available technology has enabled researchers to include larger and larger numbers of miRNA per sample, from the initial 156 miRNAs in the first study [23] to more than 1000 in the latest [24].

**Figure 1:**
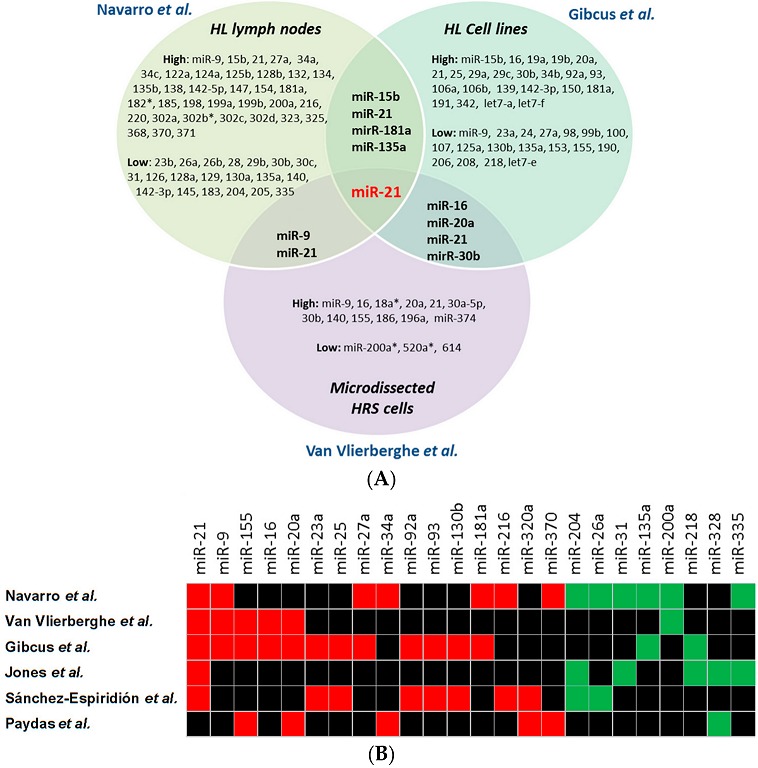
miRNA signatures in HL. (**A**) Venn diagram with the first three signatures reported in HL: one in lymph nodes [23]; one in cell lines [26]; and one in microdissected HRS cells [25]; and (**B**) overlapping miRNAs in the six HL profile studies. Only the miRNAs shared by at least two signatures and with the same expression are included. Red boxes indicate overexpressed and green underexpressed miRNAs. Black boxes indicate miRNAs not included in the list. More detailed information is included in Table 1.

Navarro et al. assessed the expression of 156 mature miRNAs in 49 lymph nodes from cHL patients and 10 reactive lymph nodes (RLNs). The unsupervised analysis showed three well-defined clusters: NS HL, MC HL, and RLNs. A 25-miRNA signature that could differentiate cHL from RLNs was identified and validated in an additional cohort of 30 cHLs and 5 RLNs, as well as in three HL cell lines (L428, L1236 and HD-MY-Z) (Figure 1). Moreover, miR-21, miR-134 and miR-138, which were part of the 25-miRNA signature, were found to be overexpressed in the cytoplasm of HRS cells by chromogenic in situ hybridization. MiRNA signatures characteristic of NS or MC subtypes of HL were also described. Twenty of the 25 miRNAs were also expressed in the three cell lines, leading the authors to speculate that these 20 miRNAs were expressed by the HRS cells while the remaining five miRNAs (miR-220, miR-302a, miR-302b, miR-302c, and miR-325) were part of the reactive microenvironment. Correlation with clinical characteristics showed that miR-138 was downregulated in Ann Arbor stages III–IV [23].

Van Vlierberghe et al. profiled 360 miRNAs in microdissected HRS cells from nine cHL patients, four HL cell lines (L1236, L540, HDLM2 and KM-H2), and CD77 + GC B cells and identified a 15-miRNA signature, including 12 upregulated and three downregulated miRNAs. miR-155 was the most expressed miRNA together with miR-21, miR-20a, miR-9 and miR-16 [25] (Figure 1).

Gibcus et al. compared the expression of 183 miRNAs in HL cell lines (L428, L1236, KM-H2, DEV) with other B cell lymphoma cell lines and described a 23-miRNA signature characteristic of HL cell lines. The signature included key overexpressed miRNAs (miR-17-92 cluster, miR-16, miR-21, miR-24, miR-155) and a downregulated miRNA (miR-150) [26] (Figure 1).

Using arrays, Sánchez-Espiridión et al. analyzed 723 human and 76 human viral miRNAs in tumor samples from 29 patients with advanced cHL and in five HL cell lines (L428, L1236, L540, HDLM2, and HD-MY-Z). They identified a 234-miRNA signature composed of 134 miRNAs upregulated in HL cell lines and 100 miRNAs upregulated in tumor samples [27].

Jones et al. examined more than 1000 miRNAs in 14 cHL tissue biopsies and in eight non-malignant lymph nodes. Only patients with NS and MC and no HIV or hepatitis B/C were included in the analysis. The analysis identified 474 miRNAs differentially expressed between cHL samples and controls; 238 miRNAs were overexpressed in cHL. The authors selected the five miRNAs with the highest expression (miR-2861, miR-638, miR-494, miR-663b and miR-1973), together with miR-155, miR-21 and miR-16 from previous works [20,23,25,26], for further study. The study by real-time PCR in the 14 cHL cases and in 26 additional samples confirmed that miR-494, miR-1973, miR-2861, miR-638, miR-21, and miR-16 were overexpressed in cHL lymph nodes, while no differences were observed for miR-155 [24].

Paydas et al. profiled 32 cHL formalin-fixed paraffin-embedded (FFPE) samples and 60 RLNs by qPCR. 377 miRNAs with a known association with cancer were studied. They reported 11 miRNAs upregulated and 13 downregulated. Two of them showed a correlation with clinic pathological variables; while miR-889 was upregulated in patients with B-symptoms, miR-127-3p was upregulated in NS compared to MC patients [28].

There is little overlap among the six miRNA signatures identified in these studies (Figure 1). Variation in the miRNAs included in the signatures may be due to the different profiling platforms, statistical analyses, and especially due to different sample types. miR-21, which is well known for its oncogenic role in solid tumors [29] and B cell lymphomas [30], was overexpressed in five out of six analyses, although it did not reach significance in the study by Jones et al. Other miRNAs shared by several signatures were miR-155, miR-9, miR-17-92 cluster, miR-204, miR-335, and miR-30b (Table 1). Interestingly, the poor overlapping between primary HRS cells and cell lines may indicate that HL-derived cell lines undergo miRNA expression changes in order to survive in cell suspension [31].

## 3. miRNAs Impacting Prognosis and Treatment Response in HL

The first miRNA to be associated with survival in cHL was miR-135a. In a cohort of 89 cHL patients, Navarro et al. found that miR-135a was downregulated in cHL lymph nodes compared to RLNs and that patients with lower levels had higher probability of relapse and shorter disease-free survival (DFS) than those with high levels. Moreover, miR-135a emerged as independent prognostic factor for DFS in the multivariate analysis [32] (Table 2).

Another study of HIV-negative patients with advanced cHL identified 34 miRNAs as differentially expressed between patients with favorable and unfavorable outcomes. The validation of 12 of these miRNAs in 168 FFPE cHL samples led to the identification of a 4-miRNA score associated with failure-free survival (FFS): miR-21, miR-92b*, miR-30d and miR-30e. Interestingly, the multivariate analysis identified the 4-miRNA score as a prognostic factor independent of International Prognostic Score (IPS) and age [27].

Jones et al. analyzed the role of seven miRNAs overexpressed in cHL lymph nodes in plasma samples from patients at three time points: pre-therapy, interim and post-therapy. Five miRNAs (miR-494, miR-2861, miR-21, miR-155, and miR-16) were significantly elevated in cHL pre-therapy in comparison with healthy controls, and moreover, high levels of these miRNAs correlated with Hasenclever scores ≥3. In addition, levels of miR-494 and miR-21, as well as miR-1973, were significantly lower in plasma from patients attaining complete remission (CR), whose levels were equivalent to those of healthy controls [24].

When miR-9, miR-20a, miR-21, miR-26a, and miR-155 were analyzed in another study in whole blood RNA from pre-therapy and post-therapy samples from HL patients, significant differences were observed in the levels of all five miRNAs after ABVD treatment, indicating that these miRNAs could be blood biomarkers of treatment response [33].

Recently, van Eijndhoven et al. [34] have demonstrated that miR-21 and miR-155 are enriched in plasma vesicles from HL patients compared with healthy controls, together with other miRNAs such as miR-24-3p, miR-127-3p, and let7a-5p. Moreover, the expression of these extracellular vesicle (EV)-associated miRNAs robustly decreased after treatment in patients with complete metabolic response (FDG-PET negative) and rose again in relapsed patients, highlighting the utility of these miRNAs as biomarkers for treatment response [34].

Of note, most of the miRNAs identified to date as having clinical or prognostic implications are shared by two or more of the six studies of miRNA signatures in HL [23,24,25,26,27,28] (Figure 1 and Table 1).

## 4. miRNAs Involved in the Pathogenesis of HL

### 4.1. miR-155

**Figure 2:**
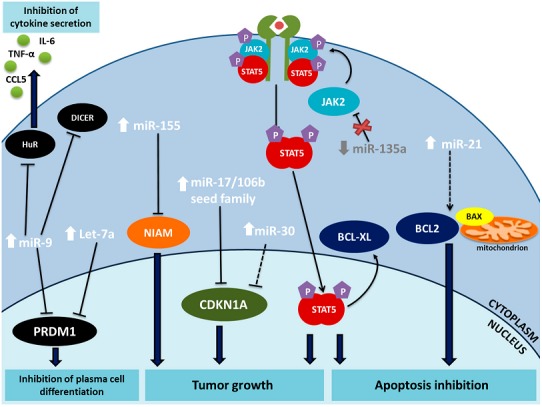
The main miRNAs involved in the pathogenesis of HL: miR-9, let-7a, miR-155, miR-17/106b seed family, miR-30, miR-135a, and miR-21. Arrows indicate the expression level of each miRNA in HL. Solid lines indicate validated regulation of the target gene, while broken lines indicate indirect regulation of the gene. Blue arrows lead from the gene to the final effect of the miRNA intervention.

In the first study associating miRNAs with HL [20], Kluiver et al. observed high levels of miR-155 in five HL cell lines, L591, L428, KM-H2, L1236 and DEV, but not in the HDLM2 cell line, which has a T cell origin. Moreover, miR-155 overexpression was identified in seven HL patients, two diffuse large B cell lymphoma patients, and one primary mediastinal B cell lymphoma patient [20]. Several studies have identified target genes for miR-155. AGTR1, ZNF537, FGF7, ZIC3, MAF, and IKBKE were identified by renilla/luciferase assay in HL cell lines [26]. Another study analyzed the targetome of miR-155 using an Ago2-RIP-Chip approach in a BL-derived cell line (ST486) with low miR-155 levels and two HL cell lines (KM-H2 and L1236) with high levels [36]. The authors identified 54 target genes in the ST486 cell line but were not able to validate targets in the HL cell lines using a miRNA sponge system. However, they selected six targets for further validation and were able to validate five in all three cell lines: DET1, NIAM, HOMEZ, PSIP1 and JARID2. NIAM inhibition by miR-155 was associated with increased proliferation of BL cells, suggesting that the oncogenic role of miR-155 in B cell lymphomas involves targeting the tumor suppressor NIAM [36] (Figure 2).

miR-155 is a key miRNA not only in HL, but also in B cell malignancies, where its ectopic expression in murine models induces lymphomagenesis [37,38]. Recently, a promising therapeutic potential for miR-155 inhibition has been observed in a miR-155-dependent mouse model of lymphoma using a nanoparticle-based delivery system [39].

### 4.2. miR-9 and let-7a

miR-9 and let-7a were identified by cloning among the ten most abundant miRNAs in L428, KMH2 and L1236 HL cell lines. Both miRNAs negatively regulated the *PRDM1/BLIMP1* gene [40], a crucial gene during B cell differentiation to plasma cells [41]. PRDM1/BLIMP1 is regulated by the transmembrane protein CD99 [42], whose downregulation in HRS cells is a key event in tumorogenesis [43] and can be induced by EBV protein LMP1 [44]. CD99 upregulation leads to a decrease in the HL markers CD30 and CD15 and to an increase in the expression of PRDM1/BLIMP1, inducing terminal B cell differentiation. CD99 upregulation also leads to a downregulation of miR-9, increasing activation of PRDM1/BLIMP1 [42] (Figure 2). Leucci et al. [45] reported that miR-9 targets DICER1, a nuclease involved in miRNAs biogenesis, and HuR (also known as ELAVL1), a protein in charge of stabilizing mRNAs. Through these targets, especially HuR, miR-9 regulates the secretion of the cytokines IL-5, IL-6, TNF-α, and CCL5, affecting the ability of HL cells to attract normal blood cells (Figure 2). Inhibition of miR-9 reduced HL tumor growth in immunodeficient NOG mice [45]. Curiously, Kuhlen et al. found that DICER1 syndrome, associated with DICER1 mutations, seems to be associated with rare forms of T cell HL [46].

### 4.3. miR-17/106b Family

miR-17/106b family (cluster miR-17~92-1, cluster miR-106b~25, and miR-106a~92-2) includes 14 miRNAs: miR-17, miR-18a, miR-19a, miR-20a, miR-19b-1, miR-92-1, miR-106b, miR-93, miR-25, miR-106a, miR-18b, miR-20b, miR-19b-2, miR-92-2, and miR-363 [47]. Tan et al. using a high throughput experimental approach to identify miRNA targets based on a RIP-Chip against Ago2 technique observed that the miRNA targetome of HL cell lines was enriched with miR-17/106b family targets. They further validated eight of the genes by Renilla-luciferase assay: YES1, RBJ, NPAT, FBX031, OBFC2A, GPR137B, CCL1, and ZNFX1 [48]. The same group reported the upregulation of several members of the miR-17/106b family in HL and the consequent downregulation of their target genes, including CDKN1A (p21 protein) that become downregulated after transfection with pre-miR-17 (Figure 2). Moreover, the downregulation of miR-17/106b family leads to a 5–10% increase in G1 arrest dependent on p21 [49].

### 4.4. miR-21 and miR-30d

Both miR-21 and miR-30d were identified by cloning as among the most highly-expressed miRNAs in HL cell lines [40]. Sánchez-Espiridión et al. reported a prognostic miR-21 and miR-30d. Moreover, they demonstrated that miR-21 and miR-30d play a role in apoptosis resistance; and inhibition of miR-21 and miR-30d increased apoptosis and sensitized the L-428 cell line to doxorubicin-induced apoptosis. Inhibition of miR-21 was associated with decreased BCL2/BAX and BCL2L1/BAX ratios in HL while miR-30d inhibition was associated with an increase of CDKN1A levels and activation of the p53 pathway [27] (Figure 2). However, these are not validated targets and can reflect indirect effects.

### 4.5. miR-135a

miR-135a was identified by our group as a tumor suppressor miRNA that impacts prognosis in HL patients. The in vitro overexpression of miR-135a led to activation of apoptosis and inhibition of proliferation. In HL cell lines, JAK2 and miR-135a presented contrasting expression patterns, where JAK2 had normal levels and miR-135a had low or no expression. When miR-135a was increased, JAK2 protein levels decreased dose-dependently. Renilla/luciferase assay confirmed that JAK2 was a target of miR-135a. Moreover, the effect of miR-135a on JAK2 correlated with a reduction of Bcl-XL mRNA, which explained the observed effect on apoptosis [32] (Figure 2). Based on these findings, our group has since studied several miRNAs potentially targeting JAK2 and validated three in addition to miR-135a: miR-101, miR-204, and miR-216b. Patients with low expression of more than two of these miRNAs had shorter overall survival (OS) [50].

### 4.6. miR-96, miR-182 and miR-183

The miR-183/-96/-182 cluster is a highly conserved polycistronic miRNA cluster encoded in chromosome 7q32.2. It is deregulated in different tumors, where it can play different roles—either as an oncogene or a tumor suppressor gene. In cHL, Navarro et al. reported that miR-96 was overexpressed in lymph nodes from EBV-negative patients. miR-182* and miR-183 were included in the miRNA signature and were overexpressed in the HL cell lines L-428 and L-1236. Xie et al. and Vogel et al. identified the FOXO1 transcription factor as a tumor suppressor gene involved in HL pathogenesis through block of plasma cell differentiation by regulating PRDM1α [51,52]. FOXO1 expression is repressed by different mechanisms in HRS cells and in HL cell lines, including the activation of ERK and AKT pathways, chromosomal deletions and the upregulation of miR-96, miR-182 and miR-183, which target FOXO1 3′UTR [51].

## 5. miRNAs Regulated by Methylation in HL

Several miRNAs have been reported to be regulated by methylation in HL. Ben Dhiab et al. assessed promoter methylation of miR-9 genes–miR-9-1 (1q22), miR-9-2 (5q14.3) and miR-9-3 (15q26.1)-in a set of 58 HL patients and observed that 84.5% of them had at least one of the miR-9 genes methylated, while none of the 15 healthy samples showed methylation [53]. These results are not in line with the previous works that have identified miR-9 as one of the most frequent overexpressed miRNAs in HRS cells and in cHL lymph nodes. The authors did not analyze the miR-9 expression in their samples to corroborate that the methylation observed really correlated with the miRNA expression. Moreover, since the authors are using lymph nodes the observed methylation could not be associated with the HRS cells.

Navarro et al. identified several methylated miRNAs by treatment with 5-aza-2-deoxycytidine (5-Aza-dC) of two HL cell lines (L428 and L1236). After treatment, 13 miRNAs were re-expressed in both cell lines but only six had a CpG island in less than 1000 bp of the miRNA promoter. MIR34A, MIR203, MIR490, MIR525, and MIR375 were methylated in both cell lines and unmethylated in control B cells, as shown by methylation specific PCR (MSP). MIR34 and MIR203 were further studied by MSP-in situ in tissue sections and were found methylated in the HRS cells [54].

miR-124a is encoded by three different loci, miR-124a-1, miR-124a-2, and miR-124a-3, all of which have CpG islands in their promoter region that can be regulated by methylation [55]. Ben Dhiab et al. reported in a cohort of 64 HL patients that all the patient lymph nodes showed aberrant methylation of miR-124a genes, while none of the 15 RLNs did. miR-124a-1 methylation correlated with a higher IPS, miR-124a-2 methylation was more frequent in men and in children, and miR-124a-3 was associated with advanced Ann Arbor stages. Having at least one of the genes methylated was associated with shorter event-free survival [53].

## 6. Genomic Changes Affecting miRNA Expression in HL

miRNA expression can be modulated by epigenetic changes and also by somatic copy number variations (CNVs), somatic nucleotide mutations, small indels, or single nucleotide polymorphisms (SNPs). It is known that HRS cells contain several gains and losses of large genomic segments and complex chromosomal defects [56], some of which can alter the number of copies of miRNAs. In 12 cHL patients rich in HRS cells, Hartmann et al. identified genomic imbalances in microdissected HRS cells by array-based comparative genomic hybridization. They identified 70 gains and 28 losses of miRNA genes in the entire set of patients. Some of these miRNA genes were also deregulated in the different miRNA signatures of HL [23,24,25,26,27,28], including the miR-30 family, miR-124, miR-196a, miR-132, miR-23a, miR-27a, and miR-17-92 clusters [57] (Table 3).

Reichel et al. performed an exome sequencing of primary HRS cells obtained by flow cytometry from ten different patients and from the cell lines L428 and L1236. Even though the study was not focused on miRNAs, they identified several miRNA genes that were affected by CNVs and indels. When they combined the results from all the samples (patients and cell lines), they found 308 miRNA genes affected by CNVs in at least three samples-126 amplified and 182 deleted. Among the miRNAs included in the six signatures [23,24,25,26,27,28] (Figure 1B), miR-16, miR-20a, miR-30b, miR-31, and miR-92 can be deregulated by CNVs [58] (Table 3).

In a recent work, Hundall et al. [59] show the results of a whole-exome sequencing and karyotypic analysis of five cHL cell lines (HDLM2, KMH2, UH01, L540, and L428). They observed that CNVs affected more than 290 miRNAs, including miR-132, miR-135a, miR-155, miR-181a, miR-196a, miR-21, miR-30 family, miR-34a, miR-9, and miR-92 [59] (Table 3).

SNPs affecting the miRNA pathway, known as miR-SNPs [60], can also modulate miRNA expression and function. In a cohort of 141 patients, Navarro et al. analyzed eight miR-SNPs [61]. They reported that miR-SNPs KRT81 rs3660 (a SNP located in the 3′UTR region of KRT81 in a miRNA binding site), TRBP rs784567 (a Dicer recruiter), XPO5 rs11077 (one of the proteins responsible for pre-miRNA transport from the nucleus to the cytoplasm), and MIR196A2 rs11614913 impacted HL treatment response and prognosis. KRT81 rs3660 and XPO5 rs11077 were associated with treatment-related neural and bleomycin-associated pulmonary toxicity, respectively. TRBP rs784567 and XPO5 rs11077 were associated with DFS. XPO5 rs11077 was also associated with OS [61].

## 7. lncRNAs in HL

LncRNAs are ncRNAs larger than 200 bp that are involved in recruitment of chromatin modification complexes, regulation of transcriptional process, enhancement and co-activation of transcription factors, silencing of gene expression, and regulation of alternative splicing (reviewed in [62]). Although some studies have examined the role of lncRNAs in hematological malignancies [63], only two studies have analyzed their impact in HL. In the cHL cell line L-428, Leucci et al. described the interaction between miR-9 and MALAT1, one of the most abundant and conserved lncRNAs. miR-9 interacts with MALAT1 via two binding sites, triggering its degradation in the nucleus in an AGO2-dependent way [64]. Evidence has linked MALAT1 to cancer development and progression [65].

Tayari et al. defined the first lncRNA profile in HL cell lines. The authors detected 9955 lncRNAs in HL cell lines (DEV, L540, SUP-HD1, L1236, KM-H2, and L428) [66]. The unsupervised hierarchical cluster analysis using the 401 lncRNA differentially expressed in GC vs. naïve and memory B cells, showed that HL cell lines had a differential expression pattern close to GC B cells. Memory and naïve B cells were found together in another cluster.

The supervised analysis identified 639 differently-expressed lncRNAs between GC B cells and HL cell lines, 74% of which were downregulated in HL. They further studied three lncRNAs (FLJ42351, LINC00116 and LINC00461) in several lymphoma cell lines by RT-qPCR and observed that all three were upregulated in HL cell lines. Moreover, RNA-FISH of the three lncRNAs in four cHL cases revealed a tumor cell-specific staining. The authors suggested that LINC00461 and FLJ42351 could act as cis-regulatory elements for MEFC2 and SLC20A1, respectively [66].

## 8. PiwiRNAs in HL

PiwiRNAs (piRNAs) are small ncRNAs larger than miRNAs, 26 to 32 bp. They are mainly involved in the regulation of chromatin and genome organization, transposon repression, and the regulation of protein synthesis (reviewed in [67]). Until very recently, they were thought to be expressed only in germinal cells, but it has since been shown that they are also expressed in somatic tissue and even in solid tumors [68,69,70] and hematological malignancies [71]. Our group reported for the first time that piRNAs and their associated Argonaute proteins, PIWI proteins, can be detected in cHL. The PIWI proteins PIWIL1, PIWIL2, and PIWIL4 were detected in HL cell lines and in the cytoplasm of HRS cells of cHL patients. The piRNAs piR-651 [68,72], piR-20365 and piR-20582 [69] were overexpressed in cHL patients in comparison with RLNs. Moreover, low levels of piR-651 correlated with shorter DFS and OS, and lack of response to first-line treatment. Interestingly, piR-651 was under-expressed in serum samples at diagnosis, but increased after a complete response to levels similar to those of healthy controls [35].

## 9. Conclusions

The study of ncRNAs in HL is still in an early developmental phase, partly owing to difficulties in studying HRS cells. The group of ncRNAs that has been most widely explored is miRNAs, which have been studied by different groups with diverse strategies. These studies have identified different miRNA signatures, including some for HL lymph nodes, one for HRS cells, and several for HL cell lines. Although the different signatures have few miRNAs in common, in part due to the different samples analyzed and the different groups compared, the identified miRNAs have been shown to play a relevant role in HL pathogenesis. They have been found to be involved in proliferation, apoptosis escape, and plasma cell differentiation. Moreover, the expression levels of some of these miRNAs have clinical utility since they can help to predict prognosis and treatment response. Some of them have been identified in blood samples and in extracellular vesicles, showing a clear value for monitoring treatment response. In summary, miRNAs have emerged as important elements in HL pathology and as useful disease markers. Other ncRNAs, such as piwiRNAs and lncRNAs, have also been explored in preliminary studies with promising results. Although a great deal of work is still required, especially to identify the value of other ncRNAs, we are confident that ncRNAs will be in the spotlight of HL pathology in the foreseeable future.

## Figures and Tables

**Table 1:** List of microRNAs present in at least two of the six miRNA signatures. Up and down arrows indicate if the miRNA is overexpressed or underexpressed in HL vs the compared group. The compared groups differ between the different studies. cHL LN: cHL lymph nodes; RLN: Reactive lymph nodes; HRS: microdissected HRS cells; CD77+ cells; HL cl: HL cell lines; BL cl: Burkit lymphoma cell lines; LCL cl: germinal center B-cell-derived lymphoblastoid cell line; CLL cl: Chronic Lymphocytic Leukemia cells; DLBCL cl: diffuse large B-cell lymphoma cell lines.

miRNAs	Navarro	Van Vlierberghe	Gibcus	Jones	Sánchez-Espiridion	Paydas
miR-9	↑ cHL LN-RLN	↑ HRS-CD77+	↑ HL cl-BL cl ^$^			
miR-15b	↓ cHL LN-RLN		↑ HL cl-BL/LCL cl			
miR-16		↑ HRS-CD77+	↑ HL cl-BL/LCL cl			
miR-20a		↑ HRS-CD77+	↑ HL cl-LCL/CLL cl			↑ cHL LN-RLN
miR-21	↑ cHL LN-RLN	↑ HRS-CD77+	↑ HL cl-BL cl	↑ cHL-RLN	↑ cHL LN-HL cl	
miR-23a			↑ HL cl-DLBCL/BL cl		↑ cHL LN-HL cl	
miR-25			↑ HL cl-BL/LCL cl		↑ cHL LN-HL cl	
miR-26a	↓ cHL LN-RLN				↓ cHL LN-HL cl	
miR-27a	↑ cHL LN-RLN		↑ HL cl-DLBCL/BL cl			
miR-29a			↑ HL cl-BL/CLL cl	↓ cHL-RLN		
miR-30b	↓ cHL LN-RLN	↑ HRS-CD77+	↑ HL cl-BL cl			
miR-31	↓ cHL LN-RLN			↓ cHL-RLN		
miR-34a	↑ cHL LN-RLN					↑ cHL LN-RLN
miR-92a			↑ HL-BL/LCL/CLL cl		↑ cHL LN-HL cl	
miR-93			↑ HL cl-BL/CLL cl		↑ cHL LN-HL cl	
miR-140	↓ cHL LN-RLN	↑ HRS-CD77+				
miR-125a	↓ cHL LN-RLN	↑ HRS-CD77+				
miR-128a	↑ cHL LN-RLN					↓ cHL LN-RLN
miR-130b			↑ HL cl-BL cl ^$^		↑ cHL LN-HL cl	
miR-132	↑ cHL LN-RLN			↓ cHL-RLN	↓ cHL LN-HL cl	
miR-135a	↓ cHL LN-RLN		↓ HL cl-PMBL cl			
miR-139			↑ HL cl-LCL cl	↓ cHL-RLN		
miR-142-3p	↓ cHL LN-RLN		↑ HL cl-BL/CLL cl			
miR-145	↑ cHL LN-RLN					↓ cHL LN-RLN
miR-155		↑ HRS-CD77+	#Non-sig.	#Non-sig.		↑ cHL LN-RLN
miR-181a	↑ cHL LN-RLN					
miR-196a		↑ HRS-CD77+		↓ cHL-RLN		
miR-200a	↓ cHL LN-RLN	↓ HRS-CD77+				
miR-204	↓ cHL LN-RLN			↓ cHL-RLN	↓ cHL LN-HL cl	
miR-216	↑ cHL LN-RLN				↑ cHL LN-HL cl	
miR-218			↓ HL cl-BL cl	↓ cHL-RLN		
miR-320a					↑ cHL LN-HL cl	↑ cHL LN-RLN
miR-328				↓ cHL-RLN		↓ cHL LN-RLN
miR-335	↓ cHL LN-RLN			↓ cHL-RLN		
miR-370	↑ cHL LN-RLN					↑ cHL LN-RLN

$ miR-9 and 130b in the Gibcus study were downregulated in the array results and upregulated in the qRT-PCR results;

#miR-155 was included in the list of abundantly expressed miRNAs although it was not found differentially expressed.

**Table 2:** Non-coding RNAs associated with clinical outcome in cHL.

ncRNAs	Levels Associated with Bad Prognosis	Samples	Patients	Endpoint	Multivariate Analysis Performed	Ref.
miR-135a	Low	Lymph nodes	89 cHL	DFS	Yes	[32]
miR-21 miR-92b-5p miR-30d miR-30e	High Low High Low	Lymph nodes	Discovery set with 29 and training set with 168 HIV-advanced cHL	Failure-free survival	Yes	[27]
miR-494 miR-21 miR-1973	High High High	Plasma	42 HIV-,HCV- and HBV-cHL	Treatment response	No	[24]
miR-9 miR-21 miR-155	High High Low	Peripheral blood	4 before treatment and 7 after treatment cHL	Treatment response	No	[33]
miR-21-5p miR-127-3-p miR-155-5p let-7a-5p	High High High High	Plasma (extracellular vesicles and protein bound associated miRNAs)	20 cHL before treatment (13 primary and 7 relapsed) and 7 after treatment	Complete metabolic response (FDG-PET)	No	[34]
piR-651	Low	Lymph nodes	94 HIV-cHL	Treatment response, DFS and OS	Yes	[35]
piR-651	Low	Serum	11 cHL before treatment and 9 after treatment	Treatment response	No	[35]

**Table 3:** Genomic changes (copy number variation) associated with miRNAs identified in at least two HL signatures. This table summarizes the data in the studies of Hartmann et al. [57], Reichel et al. [58], and Hudnall et al. [59]. Genomic location was provided using human genome GRCh38.

miRNA	Location	HL Cell Lines		HL Patients	
		Gain^+^	Loss^+^	Gain^+^	Loss^+^
miR-9	chr1: 156420341-156420429 [−] chr5: 88666853-88666939 [−] chr15: 89368017-89368106 [+]	1/2 − 2/5	− − 1/6	1/10 − −	− − 1/10
miR-15	chr3: 160404588-160404685 [+]	−	−	−	1/10
miR-16	chr13: 50048973-50049061 [−] chr3: 160404745-160404825 [+]	− −	2/2 0/2	− −	3/10 1/10
miR-20a	chr13: 91351065-91351135 [+]		2/2		2/10
miR-21	chr17: 59841266-59841337 [+]	4/5	1/5	−	−
miR-23a	chr19: 13836587-13836659 [−]	0/2	−	8/22	−
miR-25	chr7: 100093560-100093643 [−]	−	−	−	−
miR-26a	chr3: 37969404-37969480 [+] chr12: 57824609-57824692 [−]	− 1/2	1/2 1/2	− 1/10	0/10 0/10
miR-27a	chr19: 13836440-13836517 [−]	0/2	−	8/22	0/12
miR-29a	chr7: 130876747-130876810 [−]	2/5	0/6	−	2/10
miR-30b	chr8: 134800520-134800607 [−]	3/6	0/6	0/10	3/10
miR-31	chr9: 21512115-21512185 [−]	0/2	1/2	3/10	1/10
miR-34a	chr1: 9151668-9151777 [−]	4/5	0/6	−	2/10
miR-92a	chr13: 91351314-91351391 [+] chrX: 134169538-134169612 [−]	− 2/2	2/2 −	− 6/22	2/10 −
miR-93	chr7: 100093768-100093847 [−]	−	−	−	−
miR-125a	chr19: 51693254-51693339 [+]	−	−	−	−
miR-128a	chr2: 135665397-135665478 [+]	0/2	−	1/10	−
miR-130b	chr22: 21653304-21653385 [+]	1/5	1/6	4/12	4/22
miR-132	chr17: 2049908-2050008 [−]	2/5	1/6	4/12	0/10
miR-135a	chr3: 52294219-52294308 [−] chr12: 97563812-97563911 [+]	− 3/6	1/2 1/6*	− 1/10	0/10 0/10
miR-139	chr11: 72615063-72615130 [−]	−	1/2	4/12	0/10
miR-140	chr16: 69933081-69933180 [+]	−	0/2	−	1/10
miR-142-3p	chr:17 58331232-58331318 [−]	−	−	−	−
miR-145	chr5: 149430646-149430733 [+]	−	−	−	−
miR-155	chr21: 25573980-25574044 [+]	3/5	0/6	−	1/10
miR-181a	chr1: 198859044-198859153 [−] chr9: 124692442-124692551 [+]	1/2 2/6*	− 0/6	0/10 2/10	− 1/10
miR-196a	chr17: 48632490-48632559 [−] chr12: 53991738-53991847 [+]	5/5 5/6*	0/5 0/5	− 6/22	− −
miR-200a	chr1: 1167863-1167952 [+]	2/5	1/6*	−	2/10
miR-204	chr9: 70809975-70810084 [−]	0/2	0/2	1/10	1/10
miR-216	chr2: 55988950-55989059 [−]	6/6*	0/5	3/10	−
miR-218	chr4: 20528275-20528384 [+] chr5: 168768146-168768255 [−]	− 0/2	1/2 −	− 2/10	0/10 −
miR−320a	chr8: 22244962−22245043 [−]	−	1/2	−	7/22
miR-328	chr16: 67202321-67202395 [−]	−	0/2	−	1/10
miR-335	chr7: 130496111-130496204 [−]	−	0/2	−	2/10

+ The denominator represents the total number of cell lines or patients analyzed. No data available is indicated as ”–“; *Findings in the L-428 cell line differed between the studies.

## Abbreviations

HL	Hodgkin lymphoma
HRS	Hodgkin and Reed-Sternberg cells
GC	Germinal center
EBV	Epstein Barr virus
cHL	Classical Hodgkin lymphoma
NS	Nodular sclerosis
MC	Mixed cellularity
ncRNAs	Non-coding RNAs
lncRNAs	Long non-coding RNAs
miRNAs	microRNAs
XPO5	Exportin-5
RLNs	Reactive lymph nodes
FFPE	Formalin-fixed paraffin-embedded
DFS	Disease-free survival
OS	Overall survival
IPS	International Prognostic Score
CR	Complete remission
MSP	Methylation specific PCR
CNVs	Copy number variations
SNPs	Single nucleotide polymorhisms
piRNAs	piwiRNAs
